# Exploring Complementary and Alternative Medicine Use for the Management of Acne Vulgaris Among University Students: Forms, Trends, and Information Sources

**DOI:** 10.1111/jocd.16775

**Published:** 2025-01-06

**Authors:** Sari Taha, Manal Taha, Sa'ed H. Zyoud

**Affiliations:** ^1^ An‐Najah Global Health Institute An‐Najah National University Nablus Palestine; ^2^ Department of Public Health An‐Najah National University Nablus Palestine; ^3^ Médecins Sans Frontières France Nablus Palestine; ^4^ Department of Clinical and Community Pharmacy, College of Medicine and Health Sciences An‐Najah National University Nablus Palestine; ^5^ Poison Control and Drug Information Center (PCDIC), College of Medicine and Health Sciences An‐Najah National University Nablus Palestine; ^6^ Clinical Research Center An‐Najah National University Hospital Nablus Palestine

**Keywords:** acne, acne vulgaris, community health, complementary and alternative medicine, dermatology, university students

## Abstract

**Background:**

Acne vulgaris is a common skin disease that has physical and psychological impacts. Patients diagnosed with acne often use complementary and alternative medicine, despite the insufficient evidence.

**Aims:**

This cross‐sectional study sought to identify the types, trends in and reasons for utilizing complementary and alternative medicine for acne among university students.

**Patients/Methods:**

The study was conducted among health sciences students from March to June, 2023, using clinical examination and a questionnaire. All participants were assessed for a diagnosis of acne.

**Results:**

The final sample size consisted of 367 participants, of whom 68.9% were females and 31.1% were males. The study revealed a high prevalence of acne (74.4%). Among those with acne, 59.7% reported using complementary and alternative medicine, with the biological forms being most frequent at 90.2%. The reasons commonly stated for using complementary and alternative medicine were lack of severity (48.5%) and the potential for internet‐based self‐treatment (33.1%). The main sources of information were the internet (44.8%) and social media (41.1%). Age, gender, and the occurrence of acne on the chin were associated with complementary and alternative medicine use (*p* < 0.05).

**Conclusions:**

The use of complementary and alternative medicine for treating acne is widespread, with a tendency toward biological forms. Healthcare practitioners should be familiar with the patterns of complementary and alternative medicine use for the treatment of acne to better meet patient needs and concerns. Policymaking can leverage the role of online resources in addressing the use of unconventional treatment modalities.

**Abbreviations**
AV: acne vulgaris
BMI: body mass index
CAM: complementary and alternative medicine
DALY: disability‐adjusted life year
WHO: World Health Organization

## 1 Introduction

Acne vulgaris (AV) is a chronic inflammatory disease of the skin, especially in areas of rich sebaceous gland distribution. Dermatological manifestations may range from mild skin lesions, such as comedones, to severe forms of cysts and nodules, with potential long‐term scarring and pigmentation [1, 2, 3]. Moreover, AV is associated with several psychological comorbidities, such as anxiety, low self‐esteem, and body dysmorphic syndrome [4, 5, 6, 7]. Hormonal imbalance, the proinflammatory effect of *Propionibacterium acnes* , oxidant–antioxidant imbalance, and increased sebum production, and proinflammatory lipids have been implicated in the pathophysiology of AV [3, 8, 9]. The choice of treatment, including oral and topical medications, depends on the severity of AV, and patients' and physicians' preferences [10]. The burden of AV is high and continues to rise over time. In 2019, the global prevalence and the disability‐adjusted life years (DALYs) of acne were estimated at 231.2 million and 5 million, respectively [11]. Particularly, AV is a disease of adolescents and young adults, as almost 85% of these populations are affected globally [12].

Complementary and alternative medicine (CAM) has been defined differently but mostly refers to similar sets of practices. According to the MSD manual, CAM is defined as “the healing approaches and therapies that historically have not been included in conventional, mainstream Western medicine.” [13] The application of scientific criteria to test CAM practices has been challenged by methodological difficulties, including the inherent unfeasibility of standardizing treatments and outcomes, and the difficulty of blinding and designing case–control groups [14].

The use of various forms of CAM in the treatment of acne has been investigated in multiple studies, which have reported inconclusive evidence [15, 16, 17, 18, 19]. However, the use of some forms of CAM may lead to allergic, mechanical, infectious, and nutritional complications [20]. Different factors may influence patients' decision to initiate and continue using CAM in different areas, including dissatisfaction with, and failure of, conventional medicine, belief in therapeutic effectiveness, and interest in a holistic approach to health [21, 22, 23, 24].

While studies have used different definitions of CAM, its use among AV patients ranges from 52.4% to 77% [25, 26, 27, 28, 29, 30]. Some CAM practices are common regardless of the region, while others are unique to certain areas, such as the use of a locally prepared cleanser in Nigeria [31]. The commonly used CAM methods to treat AV in the Eastern Mediterranean Region (EMR) include honey, yogurt, lemon, clay, vinegar, oatmeal, in addition to other herbs and nutritional supplements [25, 28, 30, 31].

The prevalence of CAM use among Palestinians ranges between 45.9% and 91.5%. While some studies have focused on the use of herbal remedies, others have addressed CAM use among certain populations, including people with coronary heart disease, diabetes mellitus, hypertension, pediatric patients, cancer, hemodialysis, and pregnant women [32, 33, 34, 35]. However, research addressing acne in Palestine is scarce. To the best of the authors' knowledge, no research has explored the use of CAM in the management of AV in Palestine.

Patients with AV may delay or refrain from seeking medical opinion and opt for self‐medication for various reasons, including the preference for natural approaches, perceived effectiveness, and easy accessibility [26, 27, 29]. The delay in seeking medical opinion may lead to chronic complications such as scarring [2]. Similarly, some alternative treatment methods may sometime lead to adverse outcomes [20]. Therefore, exploring the forms and patterns of CAM use in the management of AV in Palestine is key to evaluating the scope of the problem and informing potential interventions. This study aimed to explore the forms of CAM utilized to manage AV and the factors influencing their utilization among university students. It also aimed to identify the patterns related to CAM use, including the reasons and sources of information related to this use.

## 2 Methods

### 2.1 Study Design

This was a cross‐sectional study based on a self‐administered questionnaire. It was conducted from March to June 2023 at the Faculty of Medicine and Health Sciences, An‐Najah National University (NNU) in Nablus, Palestine.

### 2.2 Population and Sampling

All students registered in the second semester of 2022/2023 at the Faculty of Medicine and Health Sciences, An‐Najah National University, were eligible for inclusion. Based on information obtained from the faculty administration, the population (N) of students enrolled was 5100 students, the vast majority of whom were studying medicine. To determine the minimum sample size (X), a desired margin of error (E) was set at 5%; a confidence level of 95% (the corresponding *z* value is 1.96); and the expected prevalence (P) of AV was 80.9% in a previous survey conducted among medical students at NNU [36]. The following two equations were used to determine the initial sample size (n) and to correct for a finite population (N):

$n=N/\left(1+N\;E^{2}\right)$

$X=n/1+\left(n/N\right)$

The minimum sample size was estimated at approximately 346, but the sample size was increased to 427 to account for nonresponse and missing data. Convenience sampling was utilized to invite students to participate because obtaining records of students for randomization of sampling was infeasible.

### 2.3 Data Collection Tool and Variables

All participants were assessed for acne under clear daylight by a certified physician. Each participant filled a paper‐based, self‐administered questionnaire without interaction with other participants and in front of the researcher. The questionnaire was developed by the authors in Arabic, the native language of the students. A pilot study was carried out on 25 respondents to ensure the clarity and cultural acceptability of the questionnaire. Based on the feedback, the structure and content were modified, and additional suggestions were added to the CAM list. The final questionnaire consists of three sections.

The first section gathered information on demographic characteristics, including age; gender; university major; year of study; and self‐reported weight and height to measure BMI. The World Health Organization's (WHO) BMI classification was adopted (underweight: < 18.5 kg/m^2^, normal weight: 18.5–24.9 kg/m^2^, overweight: 25.0–29.9 kg/m^2^, obese: > 30.0 kg/m^2^) [37, 38].

The second section contains clinical questions on receiving dermatologist‐prescribed treatment for AV; first‐degree family history of AV (yes, no); duration of AV (< 1 year, 1–5 years, 5–10 years, > 10 years); location of AV (forehead, right cheek, left cheek; nose; chin; and trunk); and smoking status (current, defined as daily smoking during the past year; previous, quit smoking with the specified rate > 1 year ago; or nonsmoker).

The third section contains questions that explore the use and pattern of complementary and alternative medicine. The first part of this section explored the types and forms of CAM used to manage AV and was developed by the authors based on the consultation and cross‐review of pharmacological professors who are experts in CAM, especially those forms that are used locally. Similar studies were reviewed to create a comprehensive list of regional and global CAM. The MSD Manual list of CAM was employed as a framework to assign CAM forms to the following types: biological (including animal‐ and plant‐based modalities of CAM, diet therapy, vitamins and supplements, oily products and chelation therapy); mind–body medicine (relaxation, meditation, hypnotherapy, and biofeedback‐guided imagery); whole medical systems (*Ayurveda*, homeopathy, naturopathy Chinese medicine, prophetic medicine, including *ruqyah*); manipulative and body‐based practices (*hijama*, massage, reflexology, and chiropractic); and energy medicine (acupuncture, magnets, therapeutic touch, *Reiki*) [39]. *Hijama* is a religion‐based cupping therapy that is purported to remove “stagnant blood and toxins” [40]. Most of the biological CAM therapies listed are topically applied and not orally consumed (Table 4). The second part of this section explored the patterns of CAM use, including the reasons for using CAM, sources of information, response to CAM use, and side effects associated with CAM use.

### 2.4 Data Analysis

The Statistical Package for Social Sciences program version 25 (SPSS) was used to insert and analyze the data, employing descriptive and inferential statistics. The mean (±SD) was reported for age, and percentages and frequencies were reported for categorical and ordinal variables. Percentages for potential multiple‐response variables, including acne locations, professional treatment modalities, CAM types and forms, reasons for use, sources of information, and side effects are not mutually exclusive and sum to more than 100%. The chi‐squared test was used to explore the association between the use of CAM (binary variable) and the following variables: age (categorized into 18–19, 20–21, ≥ 22 years old), gender, university major, year of study, BMI, smoking status (binary, with a smoker defined as smoking cigarettes/vape/*shisha* during the last 30 days [41]), family history, duration, bodily location of acne, and the use of professional treatment. Fisher's exact test was used when the assumptions of the chi‐squared test were violated. Significance was determined using a threshold *p* < 0.05.

### 2.5 Ethical Considerations

Permission was obtained from the IRB office at An‐Najah National University. The purposes and nature of the research study were communicated clearly to the respondents before obtaining informed consent. The privacy and confidentiality of the participants were ensured in regard to clinical information and collected data.

## 3 Results

Of the 427 students approached, 19 declined to complete the questionnaire (4.4%), and 41 were excluded from the study due to missing data (9.6%). The variables with negligible percentages of missing data were BMI (0.04%) and smoking status (0.03%). The study included a final sample of 367 participants, with 253 females (62.7%), 114 males (37.3%), and a mean age of 20.5 years (SD ± 1.54). Two‐thirds of the respondents were studying medicine (67.6%), followed by dentistry (6.8%), nursing (6.0%), and pharmacy (3.5%). Slightly below a third of the respondents were second‐year students (29.4%), followed by first‐year (24.5%), fourth‐year (16.6%), and third‐year students (15.8%). Table 1 shows the demographic characteristics of the participants in more detail.

**TABLE 1:** The demographic characteristics of the respondents.

Variable	*n* (%)
Age (years)	
18–19	116 (31.6%)
20–21	135 (36.8%)
**≥** 22	116 (31.6%)
Gender	
Male	114 (31.1%)
Female	253 (68.9%)
BMI^a^	
Underweight	32 (8.7%)
Healthy range	237 (64.6%)
Overweight	67 (18.3%)
Obese	15 (4.1%)
Study major	
Medicine	248 (67.6%)
Dentistry	25 (6.8%)
Nursing	22 (6%)
Pharmacy	13 (3.5%)
Medical imaging	12 (3.3%)
Physiotherapy	9 (2.5%)
Anesthesia and resuscitation	8 (2.2%)
Speech pathology	8 (2.2%)
Pharmacy doctor	6 (1.6%)
Medical laboratory sciences	4 (1.1%)
Midwifery	4 (1.1%)
Optometry	4 (1.1%)
Cardiac perfusion technology	3 (0.8%)
Cosmetics and skin care	1 (0.2%)
Year of study	
First year	90 (24.5%)
Second year	108 (29.4%)
Third year	58 (15.8%)
Fourth year	61 (16.6%)
Fifth year	43 (11.7%)
Sixth year	7 (1.9)
Acne history	
Yes	273 (74.4%)
No	94 (25.6%)
Smoking status^a^	
Current smoker	48 (13.1%)
Previous smoker	4 (1.1%)
Nonsmoker	303 (82.6%)

*Note:* The total values percentages of three variables (study major, year of study, and smoking status) sums up to either 100.1% or 99.9% due to rounding percentages to the nearest 0.01.

Abbreviations: AV, acne vulgaris; BMI, body mass index.

^a^ Frequency (percentages) of missing values: BMI 16 (4.3%); and 12 smoking status (3.3%).

The prevalence of AV was 74.4%, with 72.2% reporting having a family history. Of the 273 participants with AV, only 39.2% used a treatment modality prescribed by a dermatologist. The forehead was the most common area of acne (62.2%), followed by the left cheek (53.1%), right cheek (51.6%), chin (35.6%), trunk (27.4%), and nose (14.7%). For the duration of the disease, 41.0% had acne for < 1 year, 47.6% for one to 5 years, 9.5% for 5–10 years, and 1.8% for > 10 years. Table 2 includes further detail on the AV‐related clinical characteristics of respondents, and Table 3 lists the medications utilized as per professional advice.

**TABLE 2:** Association between the demographic and clinical characteristics of the respondents with acne vulgaris and the use of complementary and alternative medicine.

Variable	CAM use; *n* (%)		Total *n* ^a^	*p*
	Yes	No		
Age (years)				
18–19	62 (72.1%)	24 (27.9%)	86 (31.5%)	0.010*
20–21	56 (57.7%)	41 (42.3%)	97 (35.5%)	
**≥** 22	45 (50.0%)	45 (50.0%)	90 (33.0%)	
Gender				
Male	28 (36.8%)	48 (63.2%)	76 (27.8%)	< 0.001*
Female	135 (68.5%)	62 (31.5%)	197 (72.2%)	
BMI^b^				
Underweight	21 (80.8%)	5 (19.2%)	26 (10.0%)	0.08
Healthy range	104 (59.1%)	72 (40.9%)	176 (68.0%)	
Overweight	24 (53.3%)	21 (46.7%)	45 (17.4%)	
Obese	9 (75.0%)	3 (25.0%)	12 (4.6%)	
Family history of AV				
Yes	115 (58.4%)	82 (41.6%)	197 (72.2%)	0.47
No	48 (63.2%)	28 (36.8%)	76 (27.8%)	
Study major				
Medicine	97 (53.9%)	83 (46.1%)	180 (65.9%)	0.22
Dentistry	11 (68.8%)	5 (31.3%)	16 (5.8%)	
Nursing	14 (82.4%)	3 (17.6%)	17 (6.2%)	
Pharmacy	8 (72.7%)	3 (27.3%)	11 (4.0%)	
Medical imaging	6 (54.5%)	5 (45.5%)	11 (4.0%)	
Physiotherapy	5 (62.5%)	3 (37.5%)	8 (2.9%)	
Anesthesia and resuscitation	7 (100.0%)	0 (0.0%)	7 (2.5%)	
Speech pathology	3 (60.0%)	2 (40.0%)	5 (1.8%)	
Pharmacy doctor	3 (75.0%)	1 (25.0%)	4 (1.5%)	
Medical laboratory sciences	3 (75.0%)	1 (25.0%)	4 (1.5%)	
Midwifery	3 (75.0%)	1 (25.0%)	4 (1.5%)	
Optometry	1 (33.3%)	2 (66.7%)	3 (1.1%)	
Cardiac perfusion technology	1 (50.0%)	1 (50.0%)	2 (0.7%)	
Cosmetics and skin Care	1 (100.0%)	0 (0.0%)	1 (0.3%)	
Year of study				
First year	49 (74.2%)	17 (25.8%)	66 (24.1%)	0.08
Second year	43 (74.2%)	31 (41.9%)	74 (27.1%)	
Third year	23 (50.0%)	23 (50.0%)	46 (16.8%)	
Fourth year	25 (55.6%)	20 (44.4%)	45 (16.4%)	
Fifth year	18 (51.4%)	17 (48.6%)	35 (12.8%)	
Sixth	05 (71.4%)	02 (28.6%)	07 (2.6%)	
Duration of acne				
Less than a year	73 (65.2%)	39 (34.8%)	112 (41.0%)	0.41
1–5 years	74 (56.9%)	56 (43.1%)	130 (47.6%)	
5–10 years	13 (50.0%)	13 (50.0%)	26 (9.5%)	
> 10 years	3 (60.0%)	2 (40.0%)	5 (1.8%)	
Location of acne				
Forehead	99 (58.2%)	71 (41.8%)	170 (62.2%)	0.52
Right cheek	85 (60.3%)	56 (39.7%)	141 (51.6%)	0.90
Left cheek	83 (57.2%)	62 (42.8%)	145 (53.1%)	0.33
Nose	20 (50.0%)	20 (50.0%)	40 (14.7%)	0.16
Chin	66 (68.0%)	31 (32.0%)	97 (35.6%)	0.04*
Trunk	51 (68.0%)	24 (32.0%)	75 (27.4%)	0.09
Professional treatment				
Yes	67 (62.6%)	40 (37.4%)	107 (39.2%)	0.43
No	96 (57.8%)	70 (42.2%)	166 (60.8%)	
Smoking status				
Yes	22 (53.7%)	19 (46.3%)	41 (15.0%)	0.39
No	141 (60.8%)	91 (39.2%)	232 (85.0%)	

*Note:* The chi‐squared test was used to test the association between the use of complementary and alternative medicine and other variables. Fisher's exact test was used when the assumptions of the chi‐squared test were violated. Percentages of the locations of acne vulgaris are not mutually exclusive due to potential multiplicity of response and thus sum up to more than 100%. The total values percentages of some variables might sum up to either more or less than 100% due to rounding percentages to the nearest 0.01.

Abbreviations: AV, acne vulgaris; BMI, body mass index.

^a^ The total count is the frequency of respondents with acne (*n* = 273).

^b^ Frequency (percentages) of missing values: BMI 14 (5.1%).

* *p* value is below the threshold value for significance (0.05).

**TABLE 3:** Drugs used in cases which sought professional dermatological care.

Name of drug	*n* (%)
Facial cleanser	82 (76.6%)
Isotretinoin (PO)	52 (48.6%)
Medical cream	46 (43.0%)
Medical soap	37 (34.6%)
Adapalene and benzoyl peroxide (TOP)	4 (3.7%)
Benzoyl peroxide alone (TOP)	3 (2.8%)
Clindamycin (TOP)	3 (2.8%)
Doxycycline (PO)	3 (2.8%)
Erythromycin and Adapalene (TOP)	1 (0.9%)
Azithromycin (PO)	1 (0.9%)
Benzoyl peroxide and lipo‐hydroxy acid (TOP)	1 (0.9%)
Clindamycin and adapalene (TOP)	1 (0.9%)
Corticosteroid cream	1 (0.9%)
Fucidin (TOP)	1 (0.9%)
Salicylic acid (TOP)	1 (0.9%)
Betamethasone and Gentamicin (TOP)	1 (0.9%)

*Note:* Percentages are not mutually exclusive due to the potential multiplicity of responses and thus sum up to more than 100%.

Abbreviations: PO, per os (oral); TOP, topical.

Among those with AV, 59.7% were CAM users. Biological CAM forms were used by 90.2%, followed by mind–body practices (34.4%), physical practices (30.1%), and whole medical systems (9.8%). The most common CAM used were topically applied blossom water (by 35.6% of cases), yogurt (28.8%), aloe vera (28.4%), honey (28.2%), massage (20.9%), and rice water (17.2%), and relaxation (16.0%), lime (15.3%), meditation (11.7%), vitamin C (11.7%), multivitamins (11.0%), and *hijama* (9.8%). Table 4 lists the forms of CAM utilized by the respondents.

**TABLE 4:** Forms of complementary and alternative medicine.

CAM name	*n* (%)
Blossom water*	58 (35.6%)
Yogurt*	47 (28.8%)
*Aloe vera* *	46 (28.2%)
Honey*	44 (27.0%)
Massage	34 (20.9%)
Rice water*	28 (17.2%)
Relaxation	26 (16.0%)
Lime*	25 (15.3%)
Meditation	19 (11.7%)
*Only* Vitamin *C*	19 (11.7%)
Multivitamins	19 (11.7%)
*Hijama* (wet cupping)	16 (9.8%)
Oily products*	14 (8.6%)
Turmeric*	14 (8.6%)
Prophetic medicine	13 (8.0%)
Yoga	13 (8.0%)
Cinnamon*	13 (8.0%)
Egg*	12 (7.4%)
Yeast*	11 (6.7%)
Tea*	09 (5.5%)
Low carb diet	09 (5.5%)
Mint*	08 (4.9%)
Parsley*	08 (4.9%)
Vinegar*	07 (4.3%)
Rosemary*	06 (3.7%)
*Only* vitamin B12	06 (3.7%)
Oats*	05 (3.1%)
Only vitamin E	04 (2.5%)
Chinese medicine	03 (1.8%)
Intermittent fasting diet	03 (1.8%)
Orange*	02 (1.8%)
Georgina*	02 (1.2%)
Keto diet	01 (0.6%)
Dead sea mask*	01 (0.6%)

*Note:* Percentages are not mutually exclusive due to the potential multiplicity of responses and thus sum up to more than 100%. Vitamins were listed separately when consumed without other vitamins and as ‘multivitamins’ when consumed as part of a multivitamin‐containing product.

* These CAM modalities are topically applied and not orally consumed.

The most frequently reported reasons for using CAM were the perception that AV was not severe enough to seek professional advice (48.5%); self‐searching for treatment on the internet (33.1%); CAM was suggested by someone's recommendation (26.4%); CAM was easier to reach than professional treatment (15.3%); and the patient had no time to seek professional advice (13.5%). The most common sources of information on CAM use were the internet (44.8%), social media (41.1%), family (34.4%), friends (26.4%), healthcare professionals (20.2%), and commercials (8.6%). Table 5 shows the reasons and sources of information in more detail.

**TABLE 5:** The reasons for using complementary and alternative medicine and the source of information.

Pattern of CAM use	*n* (%)
Reasons that prompted patient to use CAM as a treatment	
Acne was not severe enough to seek professional advice	79 (48.5%)
Self‐exploration for a treatment on the internet	54 (33.1%)
Recommendations made by others	43 (26.4%)
CAM was easier to reach than medical treatment	25 (15.3%)
Limited time to seek professional advice	22 (13.5%)
Pharmacist's recommendation to use CAM	19 (11.7%)
CAM is safer compared to conventional treatment	18 (11.0%)
CAM method was effective when used previously	13 (8.0%)
High cost of conventional treatment	12 (7.4%)
Embarrassed to seek medical advice	5 (3.1%)
Failure of conventional treatment	0 (0.0%)
Source of information of using CAM	
Internet	73 (44.8%)
Social media	67 (41.1%)
Family	56 (34.4%)
Friends	43 (26.4%)
Healthcare professional	33 (20.2%)
Commercials	14 (8.6%)
Books and magazines	9 (5.5%)
Other people (not a family member or a friend)	5 (3.1%)
TV	2 (1.2%)
Articles	2 (1.2%)

*Note:* Percentages are not mutually exclusive due to the potential multiplicity of responses and thus sum up to more than 100%.

Less than half of CAM users reported some benefit from using CAM (43.5%), and slightly over a third reported no effect (38.0%), while only an insignificant minority said their acne worsened after using CAM (0.01%). The most common side effects of CAM were dehydration (30.7%), erythema (28.2%), desquamation (24.5%), skin itching (14.1%), eye itching (6.7%), eye redness (6.7%), and skin discoloration (4.9%). Table 6 shows the reported responses to CAM and side effects in more detail.

**TABLE 6:** Results of using complementary and alternative medicine.

Pattern of CAM use	*n* (%)
The response to using CAM	
Better effect	71 (43.5%)
Worse effect	03 (0.01%)
No effect	62 (38.0%)
Side effects of CAM	
Dehydration	50 (30.7%)
Erythema	46 (28.2%)
Desquamation	40 (24.5%)
Skin itching	23 (14.1%)
Eye itching	11 (6.7%)
Eye redness	11 (6.7%)
Skin discoloration	8 (4.9%)
Swelling	7 (4.3%)
Nail changes	4 (2.5%)
Hair changes	4 (2.5%)
Ulcers	4 (2.5%)
Fever	3 (1.8%)
Scaling	1 (0.6%)

*Note:* Percentages are not mutually exclusive due to the potential multiplicity of responses and thus sum up to more than 100%.

Only age (*p* = 0.010), gender (*p* < 0.001), and having acne on the chin (*p = 0*.04) were significantly associated with using CAM. Data analysis did not reveal any significant association between using CAM and any other demographic and clinical variables, including university major (*p = 0*.01), year of study (*p* = 0.08), BMI (*p* = 0.08), smoking status (*p = 0*.39), family history of acne (*p = 0*.47), duration of acne (*p* = 0.418), receiving dermatologist‐prescribed treatment (*p* = 0.44), and all other body locations of acne (*p > 0*.05). Table 2 displays the associations in more detail.

## 4 Discussion

The use of unconventional treatment methods in the management of AV may be laden with serious complications. To the best of the authors' knowledge, this was the first study to explore the use of CAM in the management of AV in Palestine. The study used a self‐administered questionnaire to identify the methods of CAM used to manage AV and the factors influencing their use among students in health‐related sciences. This study revealed a high prevalence of AV among the respondents, who used a variety of CAM forms, especially biologically based preparations, for various reasons. This use was associated with gender, age, and having acne on the chin. The most commonly cited sources of information to use CAM were the internet, social media, and family.

This study revealed that the prevalence of AV was 74.4%. In comparison with other studies among similar age groups, the prevalence reported by this study is higher than that in China (51.3%), Saudi Arabia (56.2%), France (60.7%), and Turkey (65.4%) [28, 42, 43, 44] but lower than that in Brazil and another study in Saudi Arabia [29, 45]. The global prevalence of AV varies widely, particularly across age groups, with the highest incidence in adolescence [46]. Variability in the prevalence of AV among young adults can be attributed to differences in nutritional habits and methodology, including demographic characteristics of populations, sample size, and data collection tools [46, 47, 48]. For instance, AV can be self‐reported or diagnosed by a clinician, potentially leading to over‐ or underestimation of the actual prevalence.

Most of the participants who have AV used at least one form of CAM (59.7%), with most using biological CAM. The popularity of CAM among diverse patient populations in Palestine can be ascribed to the influence of culture and religion. These traditional healing practices, rooted in Palestinian culture and transmitted across generations, embody a holistic and proactive approach to well‐being that promotes the use of CAM. Moreover, religious beliefs not only encourage the use of spiritual practices but also promote nonspiritual methods, such as herbs and other botanical remedies, as mentioned frequently in religious texts [49, 50].

However, the prevalence of CAM utilization among university students with AV is lower than the rates reported in most local studies involving other populations, including pregnant women, pediatric patients, and patients with hypertension and cancer [32, 33, 34, 35, 51]. This discrepancy might be attributed to differences in the clinical and demographic characteristics of the study population. Pregnant women, for example, are more inclined to comply with professional advice that emphasizes the intake of vitamins and minerals and discourages the use of herbs, aligning with pregnancy‐specific safety concerns [34]. Furthermore, patients with hypertension and cancer tend to be older than university students and are thus more inclined toward traditional medicine [32, 51]. In parallel, a study that investigated CAM use among university students, who have similar characteristics to the current study population, revealed reasonable use of CAM compared with older populations [52]. Moreover, students studying health‐related majors may be less likely to use CAM because of the knowledge, awareness, and culture emphasizing adherence to conventional biomedicine.

In comparison with global studies conducted among patients with AV, the rate of CAM use in this study is comparable to findings from two studies in Turkey (56.9% and 64.3%) [27, 28] but lower than those reported in three other studies in Turkey (74.41%), Saudi Arabia (77%), and South Korea (87.4%) [25, 30, 53]. This variation may have arisen from differences in methodology; for instance, one study included facial cleansers, medical soaps, and medical creams within the definition of CAM, although these are regularly prescribed by dermatologists as part of the treatment regimen [53].

The most frequently used type of CAM in this study was biological, with topically applied blossom water, yogurt, aloe vera, honey, massage, and rice water identified as the most common forms. Some of these animal‐ or plant‐based products are also used regionally to varying degrees to manage AV. Similar to the present study, honey, yogurt, and lemon were the most commonly used CAM in Saudi Arabia [25, 26], while herbs, lime, clay, blossom water, and vinegar were more commonly used in Turkey [27, 30]. The prevailing CAM methods used for AV were substantially different in other global regions. In South Korea, one study reported that cosmetics, diet therapy, and cleansing and bathing techniques were most common [53]. In Nigeria, a locally made cleanser, *Dudu Osun*, was commonly used [31]. The increasing variation in CAM forms with the movement from similar ethnocultural to regional and global settings highlights the impact of culture and religion on the pattern of CAM use. In Palestine, certain biological CAM, such as herbs and honey, are commonly used among the most studied populations, as these are rooted in inherited cultural practices and encouraged by religious texts. On the other hand, energy‐based practices and whole therapeutic systems, such as Chinese medicine and Reiki, are unpopular and thus rarely used, except for religion‐based practices such as prophetic medicine and *hujama*, as revealed by this study. Moreover, blossom water and aloe vera, which were commonly reported in the present study, are components of many over‐the‐counter acne preparations worldwide. Research into the efficacy of aloe vera in treating skin conditions has been undertaken, but more extensive research is still needed [54, 55].

The most reported reasons for using CAM in this study were the perception that AV was not severe enough to seek medical opinion, the influence of the internet and people, and the convenience associated with using CAM. This is unlike other regional studies in which reasons for using CAM were related to the ineffectiveness of medical therapy, recurrence reduction, affordability, and safety [30, 56]. Only in one other study did the lack of severity encourage the participants to self‐medicate, although not necessarily with CAM [26]. Given that most cases of AV in the present study were mild, patients might have found it cumbersome to seek medical opinion, especially in an environment conducive to using CAM. The relative inconvenience associated with seeking medical opinion might also be attributed to the loaded appointment schedule and shortage of Palestinian dermatologists, with 1.13 dermatologists per 100 000 individuals [57].

In this study, the internet and social media were the most cited sources of information regarding CAM, replacing traditional influencers, such as family and friends, followed by healthcare professionals. Similarly, the internet was the major source of information in other studies conducted among AV patients in South Korea and Turkey [30, 53], while family and friends were still the main influencers in Saudi Arabia [56]. The growing influence of social media and the digital world on health‐seeking behavior [58], especially on the use of unorthodox medicine, underscores the need for tailored interventions to address this trend and ensure access to reliable information, especially among females who were found to significantly use more CAM. Moreover, patients may turn to digital resources when CAM is not discussed in clinical settings. Integrating CAM knowledge into the medical education and clinical practice of medical students and physicians, along with encouraging discussions about CAM in clinical settings, is key for improving care quality and ensuring patient safety. Notably, specialty programs around the world have been increasingly adopting CAM‐integrated curricula in their continuous medical education [59, 60, 61, 62].

This study showed that gender and age are significantly associated with CAM. The gender association is consistent with other regional studies involving AV patients [25, 26, 27, 30, 56]. This might be explained by the gender roles of females as more involved in skincare and beauty practices [63]. Likewise, global studies have demonstrated that females are more inclined to resort to CAM use in general, which was shown to be affected by social factors such as income level [64, 65, 66]. Contrary to our study, the age association was not reported in other studies conducted among wider age groups, limiting comparison with this study [25, 27, 30]. Only one study reported such an association among university students, but it focused on over‐the‐counter medications [26]. Of note, seeking a professional opinion was not associated with CAM use in the present study. This is in contrast to a Korean study where AV patients who sought a medical opinion were significantly less likely to use traditional medicine [67]. This contrast may indicate healthcare workers' reluctance to discuss CAM in local dermatology practice, which further stresses the need to design interventions targeting healthcare workers and incorporating CAM into clinical practice.

## 5 Strengths and Limitations

This is the first study to measure the prevalence of AV in among Palestinian young adults, who are a typical age group affected by AV. This helps reflects the scale of the problem and forms the basis for further research to evaluate the economic burden of AV and guide healthcare resource allocation and health policy planning. However, this study may exhibit measurement bias for several reasons. First, the utilization of self‐reported questionnaires for data collection introduces the potential for reporting bias and recall bias. Respondents might have provided dishonest responses due to social desirability concerns. They might have also been unaware of certain CAM products, particularly those that are prepared by others or commercially manufactured. Moreover, the compilation of CAM practices in the survey may not encompass the full spectrum of these practices, as the selection is profoundly influenced by cultural factors. Although respondents were given the opportunity to supplement the provided list with unlisted CAM methods, the ability to do so was constrained by difficulties in recalling and comprehending the defining characteristics of CAM methods. In addition, the reported side effects were not matched to a CAM form or period of CAM use. Moreover, specific types of CAM, such as biological options, were more commonly used by the participants than other types, such as whole medical system, limiting generalizability to certain types of CAM. Finally, the study reliance on a nonprobability convenience sampling technique and its exclusive focus on data collection within a single university context limit its generalizability. As such, caution must be exercised when extrapolating the findings to the broader population of Palestinian university students.

## 6 Conclusions

AV is a prevalent dermatological disease that may lead to chronic scarring and psychological comorbidity. The use of CAM to manage AV is common despite the inconclusive evidence. This study aimed to investigate the use of CAM in the management of AV, including the forms and patterns of CAM use and the factors influencing this usage. The majority of university students used CAM, which was associated with age and gender. Most used biological forms of CAM due to reasons related to lack of severity, self‐exploration on the internet, and the influence of others. The internet and social media, followed by family and friends, were the most common sources of information. Interventions should address the emerging influence of the internet and social media on the use of unconventional treatment modalities, especially among females. Moreover, CAM should be integrated into medical education and clinical practice of physicians to address possible CAM use among AV patients and its potential consequences.

## Author Contributions

S.T. performed the literature search, collected and analyzed the data, and drafted the manuscript. M.T. was responsible for the integrity of the data and results, participated in the writing of the manuscript and data analysis, and provided critical input to improve the intellectual content of the study. S.T. and S.H.Z. established the concept and design, supervised the survey team, led the data analysis, and contributed to the final writing of the manuscript. All authors read and accepted the final manuscript.

## Ethics Statement

The *Institutional Review Board* (*IRB*) *of An‐Najah National University* approved this study. They issued the appropriate permission documents for it. The students were free to accept or reject the invitation to participate in the study voluntarily. Verbal informed consent was obtained from each subject who agreed to participate in this study. The confidentiality of the data was ensured. The *IRB of An‐Najah National University* approved only verbal informed consent. The reason for verbal informed consent is that participants were only required for the interview and were not subjected to any harm as long as their privacy was kept confidential. The authors confirmed that all the methods were performed following the relevant guidelines and regulations.

## Conflicts of Interest

The authors declare no conflicts of interest.

## Data Availability

Data collected and analyzed for this study are available from the corresponding author upon reasonable request.
